# Hyperuricemia in acute gastroenteritis is caused by decreased urate excretion *via* ABCG2

**DOI:** 10.1038/srep31003

**Published:** 2016-08-30

**Authors:** Hirotaka Matsuo, Tomoyuki Tsunoda, Keiko Ooyama, Masayuki Sakiyama, Tsuyoshi Sogo, Tappei Takada, Akio Nakashima, Akiyoshi Nakayama, Makoto Kawaguchi, Toshihide Higashino, Kenji Wakai, Hiroshi Ooyama, Ryota Hokari, Hiroshi Suzuki, Kimiyoshi Ichida, Ayano Inui, Shin Fujimori, Nariyoshi Shinomiya

**Affiliations:** 1 Department of Integrative Physiology and Bio-Nano Medicine, National Defense Medical College, Tokorozawa, Saitama 359-8513, Japan; 2 Department of Pediatric Hepatology and Gastroenterology, Saiseikai Yokohamashi Tobu Hospital, Yokohama, Kanagawa 230-0012, Japan; 3 Ryougoku East Gate Clinic, Sumida-ku, Tokyo 130-0026, Japan; 4 Department of Dermatology, National Defense Medical College, Tokorozawa, Saitama 359-8513, Japan; 5 Department of Pharmacy, The University of Tokyo Hospital, Bunkyo-ku, Tokyo 113-8655, Japan; 6 Division of Kidney and Hypertension, Department of Internal Medicine, Jikei University School of Medicine, Minato-ku, Tokyo 105-8471, Japan; 7 Department of Urology, National Defense Medical College, Tokorozawa, Saitama 359-8513, Japan; 8 Department of Preventive Medicine, Nagoya University Graduate School of Medicine, Nagoya, Aichi 461-8673, Japan; 9 Department of Internal Medicine, National Defense Medical College, Tokorozawa, Saitama 359-8513, Japan; 10 Department of Pathophysiology, Tokyo University of Pharmacy and Life Sciences, Hachioji, Tokyo 192-0392, Japan; 11 Department of Internal Medicine, Teikyo University School of Medicine, Itabashi-ku, Tokyo 173-8605, Japan

## Abstract

To clarify the physiological and pathophysiological roles of intestinal urate excretion *via* ABCG2 in humans, we genotyped *ABCG2* dysfunctional common variants, Q126X (rs72552713) and Q141K (rs2231142), in end-stage renal disease (hemodialysis) and acute gastroenteritis patients, respectively. ABCG2 dysfunction markedly increased serum uric acid (SUA) levels in 106 hemodialysis patients (*P* = 1.1 × 10^−4^), which demonstrated the physiological role of ABCG2 for intestinal urate excretion because their urate excretion almost depends on intestinal excretion *via* ABCG2. Also, ABCG2 dysfunction significantly elevated SUA in 67 acute gastroenteritis patients (*P* = 6.3 × 10^−3^) regardless of the degree of dehydration, which demonstrated the pathophysiological role of ABCG2 in acute gastroenteritis. These findings for the first time show ABCG2-mediated intestinal urate excretion in humans, and indicates the physiological and pathophysiological importance of intestinal epithelium as an excretion pathway besides an absorption pathway. Furthermore, increased SUA could be a useful marker not only for dehydration but also epithelial impairment of intestine.

Hyperuricemia is a common disease which induces gout, and can lead to renal disorder, hypertension, cardiovascular or cerebrovascular diseases^1^. ATP-binding cassette transporter, subfamily G, member 2 (*ABCG2/BCRP*) is a high-capacity urate transporter^2^ and expresses in both intestine^3^ and kidney^4^. We and others previously demonstrated that ABCG2 dysfunction by its common variants causes gout^2^,^5^,^6^ and hyperuricemia^2^,^7^ by decreasing urate excretion. However, the evaluation of intestinal urate excretion in humans is very difficult due to urate degradation by intestinal bacterial flora. Thus, our previous study^8^ has revealed the importance of ABCG2 for intestinal urate excretion using *Abcg2*-knockout mice, but not in humans. In this study, to clarify the physiological role of intestinal urate excretion *via* ABCG2 in humans, we performed genotyping of *ABCG2* dysfunctional variants in end-stage renal disease (hemodialysis) patients whose serum uric acid (SUA) levels are extremely elevated^9^,^10^ and urate excretion almost depends on intestinal excretion *via* ABCG2 because of their almost complete absence of renal urate excretion. Furthermore, to investigate the pathophysiological role of intestinal urate excretion *via* ABCG2 in intestinal diseases, we also performed genotyping of *ABCG2* dysfunctional variants in acute gastroenteritis patients whose ABCG2 function of intestinal urate excretion should be seriously impaired due to damage to the intestinal epithelium.

## Results

### Genotyping of *ABCG2*

Genotyping results of the two *ABCG2* dysfunctional variants, Q126X (rs72552713) and Q141K (rs2231142), for 106 hemodialysis patients, 106 sex- and body mass index (BMI)-matched health examination participants and 67 acute gastroenteritis patients, were shown in Table 1. The call rates for both variants were 100%, and they were in Hardy-Weinberg equilibrium (*P* > 0.05). Haplotype frequency of Q126X and Q141K was estimated as shown in Supplementary Table 1. This result indicates that there is no simultaneous presence of the minor allele of Q126X (“T” allele) and Q141K (“A” allele) in one haplotype, which is consistent with our previous study^2^. Therefore, we presumed the diplotypes of all samples as shown in Table 1. In this study, all of the participants were divided into three groups (full function, 3/4 function and ≤1/2 function) based on estimated ABCG2 function for the following analyses.

### Analysis of hemodialysis patients

The estimated ABCG2 function of 106 hemodialysis patients and the mean SUA for each group were shown in Table 2. The less activity the ABCG2 function showed the higher the SUA (7.1 mg/dl for full function, 7.9 mg/dl for 3/4 function and 8.4 mg/dl for ≤1/2 function), and multiple regression analysis revealed that ABCG2 dysfunction significantly elevated SUA (*P* = 1.1 × 10^−4^). On the other hand, in 106 sex- and BMI-matched health examination participants, ABCG2 dysfunction tended to elevate SUA (5.3 mg/dl for full function, 5.0 mg/dl for 3/4 function and 6.0 mg/dl for ≤1/2 function), although not significantly (*P* = 0.36, Table 2).

### Analysis of acute gastroenteritis patients

The SUA levels of 67 patients were measured during an acute period of gastroenteritis. Additionally, the SUA levels of 55 patients were measured during the recovery period from gastroenteritis. The mean SUA levels of the acute and recovery period (Table 2) were 8.8 mg/dl and 4.7 mg/dl, respectively, and the paired *t*-test showed a significant difference between them (*P* = 2.3 × 10^−12^). The number of patients, who were divided into three groups by estimated ABCG2 function, and the mean SUA levels at the acute and recovery period of gastroenteritis were shown in Table 2. In the acute period, ABCG2 dysfunction significantly elevated SUA (7.5 mg/dl for full function, 9.6 mg/dl for 3/4 function and 10.6 mg/dl for ≤1/2 function, *P* = 6.3 × 10^−3^), and the degree of dehydration also affected SUA (*P* = 1.6 × 10^−3^, Supplementary Table 2). However, ABCG2 dysfunction was not associated with the degree of dehydration in the acute period (*P* = 0.50, Table 3) and the significant association between ABCG2 dysfunction and SUA remained after the adjustment for the degree of dehydration (*P* = 7.8 × 10^−3^), indicating that the association between ABCG2 dysfunction and SUA was not due to dehydration. Regarding the recovery period, there was a trend for SUA to increase by ABCG2 dysfunction (4.2 mg/dl for full function, 4.9 mg/dl for 3/4 function and 5.4 mg/dl for ≤1/2 function, Table 2), but it was not significant (*P* = 0.10).

## Discussion

**Figure 1:**
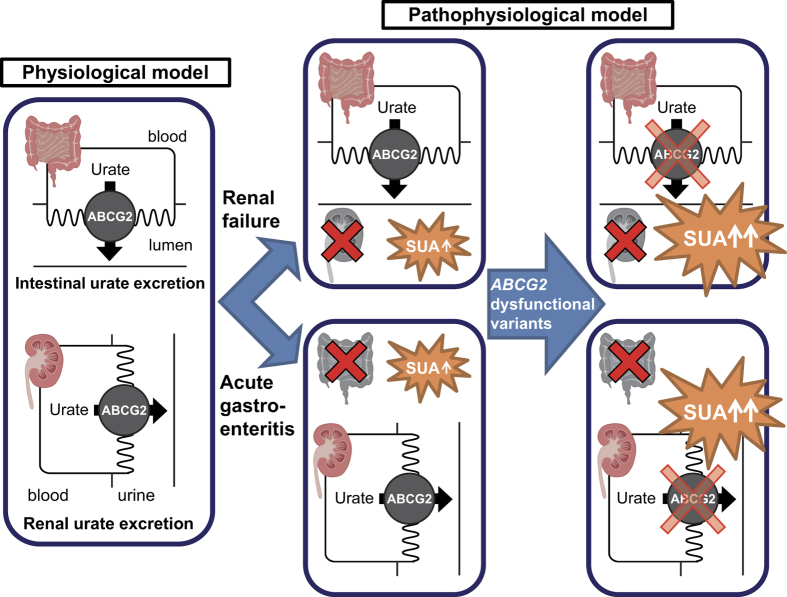
Pathophysiological model of ABCG2-mediated urate excretion in end-stage renal disease and acute gastroenteritis patients. SUA, serum uric acid. ABCG2 physiologically mediates urate excretion in both intestine and kidney. In end-stage renal disease (renal failure) patients, renal urate excretion would be nearly eliminated with urate excretion depending almost entirely on intestinal excretion. Thus, the degree of intestinal ABCG2 dysfunction strongly affects the severity of hyperuricemia in renal diseases such as end-stage renal disease. On the other hand, in acute gastroenteritis patients, intestinal inflammation seriously impairs the intestinal urate excretion *via* ABCG2. Therefore, the degree of renal ABCG2 dysfunction markedly affects the severity of hyperuricemia in intestinal diseases such as acute gastroenteritis patients.

ABCG2, which mediates urate excretion, expresses in both intestine^3^ and kidney^4^. About two-thirds of urate is excreted from kidney and about one-third from intestine^11^,^12^. This is consistent with our previous study using *Abcg2*-knockout mice^8^. However, ABCG2-mediated intestinal urate excretion has not been directly shown by human study. In end-stage renal disease (hemodialysis) patients whose SUA levels are extremely elevated^9^,^10^, renal urate excretion is nearly completely absent, and almost all urate excretion must depend on intestinal excretion *via* ABCG2. Thus, it was supposed that the degree of intestinal ABCG2 dysfunction strongly affects the severity of hyperuricemia in hemodialysis patients (Fig. 1), as was shown by multiple regression analysis in the present study (Table 2). This finding is the first evidence for a physiological role of ABCG2 on intestinal urate excretion in humans.

Besides the physiological role for intestinal urate excretion *via* ABCG2 in humans, we for the first time demonstrated that hyperuricemia in acute gastroenteritis patients is caused by decreased urate excretion in addition to dehydration which is generally considered to be a major cause of hyperuricemia in acute gastroenteritis patients^13^. Pathogens which cause acute gastroenteritis, such as rotaviruses, primarily infect the villus epithelium of the small intestine^14^,^15^,^16^,^17^. These viruses induce the destruction of infected intestinal epithelial cells, but they also mediate the down-regulation of the expression of absorptive enzymes, transporters and cytokines, which instigate malabsorption of D-xylose, lipid or lactose^14^,^17^,^18^. In acute gastroenteritis patients, intestinal inflammation would also seriously impair the function of intestinal urate excretion of ABCG2, which could be one of the reasons why SUA is markedly increased in acute gastroenteritis patients. Therefore, it is clearly possible that the degree of renal ABCG2 dysfunction affects the severity of hyperuricemia in gastroenteritis patients (Fig. 1), as was first shown by linear regression analysis in acute period gastroenteritis patients in the present study (Table 2).

The evaluation of intestinal urate excretion in humans is very difficult because urate excreted into the intestinal lumen is rapidly metabolized by bacterial flora. Thus, our previous study^8^ could reveal the importance of ABCG2 for intestinal urate excretion not using human, but rather *Abcg2*-knockout mice treated with oxonate, an uricase inhibitor. In addition, another study has also reported the decreased intestinal excretion and increased plasma concentration of uric acid in *Abcg2*-knockout mice^19^.

Taking into account the results from both hemodialysis and acute gastroenteritis patients in the present study, we for the first time demonstrated that ABCG2 mediates intestinal urate excretion in humans, which suggests the physiological importance of intestinal epithelium as an excretion pathway besides an absorption pathway. In addition, if an end-stage renal disease patient develops acute gastroenteritis, both renal and intestinal urate excretion *via* ABCG2 will extremely decrease, and thereby greatly elevate SUA.

In light of these findings, although further studies would be necessary because of the limited sample size in this study, we proposed a physiological model of urate excretion *via* ABCG2 in humans, and a pathophysiological model of hyperuricemia in intestinal and renal diseases (Fig. 1). Physiologically, ABCG2 mediates urate excretion in both intestine and kidney in humans. Pathophysiologically, in end-stage renal disease patients, the degree of intestinal ABCG2 dysfunction strongly affects the severity of hyperuricemia because urate excretion almost all depends on intestinal excretion *via* ABCG2. Contrarily, in acute gastroenteritis patients, the function of intestinal urate excretion *via* ABCG2 is severely impaired. Therefore, the degree of renal ABCG2 dysfunction clearly affects the severity of hyperuricemia. By this proposed model, physicians will recognize that increased SUA levels could be a useful marker not only for dehydration but also for intestinal impairment which induces urate export failure in intestines. Physicians could also consider “the urate excretion failure due to intestinal impairment” as one of the common causes of hyperuricemia which is often complicated in patients with acute gastroenteritis.

In summary, we revealed that two common dysfunctional variants (Q126X and Q141K) of *ABCG2* have a significant negative effect on both intestinal and renal urate excretion in humans, and that intestinal and renal ABCG2 dysfunction markedly increases SUA in end-stage renal disease and acute gastroenteritis. These findings for the first time demonstrated the physiological and pathophysiological roles of ABCG2 on intestinal urate excretion in humans.

## Methods

### Participants

This study was approved by the institutional ethical committee of the National Defense Medical College, and all procedures were performed in accordance with the Declaration of Helsinki with written informed consent from each subject. When the participant was a minor, written informed consent was obtained from each parent or guardian of that participant. Degree of dehydration in acute gastroenteritis patients was evaluated by physicians (T. Tsunoda and T.S.) according to the criteria recommended by the Center for Disease Control (CDC)^20^, and classified as “minimal or no dehydration”, “mild to moderate dehydration”, and “severe dehydration”.

In order to clarify the physiological role of intestinal urate excretion *via* ABCG2, 106 maintenance hemodialysis patients not taking medications for hyperuricemia were assigned from among the outpatients at Ryougoku East Gate Clinic (Tokyo, Japan). Their SUA levels were measured three times just before each maintenance hemodialysis, and the average was used for analyses. In addition, 106 sex- and BMI-matched subjects were selected from health examination participants in the Shizuoka area in the Japan Multi-Institutional Collaborative Cohort Study (J-MICC Study)^21^,^22^.

Sixty-seven pediatric patients with acute gastroenteritis were also recruited at the Department of Pediatric Hepatology and Gastroenterology in Saiseikai Yokohamashi Tobu Hospital (Yokohama, Japan). Their SUA levels were measured twice at the acute and recovery period of gastroenteritis.

The details of participants in this study are shown in Supplementary Table 3.

### Genetic analysis and estimation of ABCG2 function

Genomic DNA was extracted from whole peripheral blood cells^23^. Genotyping of *ABCG2* dysfunctional variants, Q126X (rs72552713) and Q141K (rs2231142), was performed using the TaqMan method (Life Technologies Corporation, Carlsbad, CA, USA) with a LightCycler 480 (Roche Diagnostics, Mannheim, Germany) as previously described^24^. Custom TaqMan assay probes were designed as follows: for Q126X, VIC-CCACTAATACTTACTTGTACCAC and FAM-CCACTAATACTTACTTATACCAC; for Q141K, VIC-CTGCTGAGAACTGTAAGTT and FAM-CTGCTGAGAACTTTAAGTT. To confirm their genotypes, DNA sequencing analysis was performed with the following primers: for Q126X, forward 5′-TGTACAATGAAAAGAGAAAGGTGAG-3′ and reverse 5′-CTGCCTTTTCACATAAGTGTC-3′; for Q141K, forward 5′-ATGGAGTTAACTGTCATTTGC-3′ and reverse 5′-CACGTTCATATTATGTAACAAGCC-3′. Direct sequencing was performed with a 3130xl Genetic Analyzer (Life Technologies Corporation)^23^,^24^.

We previously reported that Q126X is a nonfunctional variant, Q141K is a half-functional variant for urate excretion compared to the wild-type, and that there was no simultaneous presence of the minor alleles of Q126X and Q141K in one haplotype^2^, which is confirmed in the participants of the present study (Supplementary Table 1). Thus, three haplotypes were defined as *1 (126Q and 141Q), *2 (126Q and 141K) and *3 (126X and 141Q) as previously reported^25^, and all patients could be divided into the following ABCG2 functional groups: full function (*1/*1), 3/4 function (mild dysfunction, *1/*2), 1/2 function (moderate dysfunction, *1/*3 or *2/*2), and ≤1/4 function (severe dysfunction, *2/*3 or *3/*3)^25^ as shown in Table 1.

### Statistical analysis

For all calculations in the statistical analysis, the software R (version 3.1.1) (http://www.r-project.org/) was used^26^. Comparison of SUA between the acute and recovery period of gastroenteritis was performed with a paired *t*-test using a two-tailed *P* value. Linear regression analysis was performed to test the hypothesis that there was no relation between ABCG2 dysfunction and SUA in the analysis of acute gastroenteritis patients. Multiple regression analysis including ABCG2 function and age in the model was used for the analysis of hemodialysis patients and sex- and BMI-matched health examination participants, because age could not be completely matched in the selection from health examination participants. The association between ABCG2 and dehydration was examined using the Cochran-Armitage trend test. Haplotype estimation was performed with the EM algorithm^27^ using the package haplo.stats of the software R. We set the significance threshold as α = 0.05.

## Additional Information

**How to cite this article**: Matsuo, H. *et al*. Hyperuricemia in acute gastroenteritis is caused by decreased urate excretion *via* ABCG2. *Sci. Rep*. **6**, 31003; doi: 10.1038/srep31003 (2016).

## Supplementary Material

Supplementary Information

## Figures and Tables

**Table 1:** Genotyping results for each estimated ABCG2 function of participants.

Estimated ABCG2 function	Rs72552713 (Q126X)	Rs2231142 (Q141K)	Diplotype*	Number of participants		
				Hemodialysis	Health examination†	Acute gastroenteritis
Full function	C/C	C/C	*1/*1	51	50	29
3/4 function	C/C	C/A	*1/*2	46	41	30
1/2 function	C/C	A/A	*2/*2	4	8	7
	C/T	C/C	*1/*3	3	5	1
≤1/4 function	C/T	A/C	*2/*3	2	2	0
	T/T	C/C	*3/*3	0	0	0
Total				106	106	67

^*^*1, *2 and *3 represent haplotypes “C-C” (126Q and 141Q), “C-A” (126Q and 141K) and “T-C” (126X and 141Q) of two dysfunctional variants, Q126X (rs72552713) and Q141K (rs2231142), respectively.

^†^106 health examination participants were matched for sex- and body-mass index to 106 hemodialysis patients and selected from J-MICC Study.

**Table 2:** Estimated ABCG2 function and SUA of hemodialysis patients and acute gastroenteritis patients.

Estimated ABCG2 function (Diplotype of Q126X and Q141K)*	Hemodialysis						Acute gastroenteritis					
	Case			Control†			Acute period			Recovery period		
	N	SUA (mg/dl)	β (SEM)‡ *P* value§	N	SUA (mg/dl)	β (SEM)‡ *P* value§	N	SUA (mg/dl)	β (SEM)‡ *P* value||	N	SUA (mg/dl)	β (SEM)‡ *P* value||
Full function (*1/*1)	51	7.1 ± 0.1		50	5.3 ± 0.2		29	7.5 ± 0.5		24	4.2 ± 0.3	
3/4 function (*1/*2)	46	7.9 ± 0.1		41	5.0 ± 0.2		30	9.6 ± 0.7		24	4.9 ± 0.4	
≤1/2 function (*1/*3, *2/*2, *2/*3 or *3/*3)	9	8.4 ± 0.7		15	6.0 ± 0.3		8	10.6 ± 1.4		7	5.4 ± 0.6	
Total	106	7.5 ± 0.1	0.63 (0.16) *P* = 1.1 × 10^−4^	106	5.3 ± 0.1	0.17 (0.18) *P* = 0.36	67	8.8 ± 0.4	1.73 (0.61) *P* = 6.3 × 10^−3^	55	4.7 ± 0.2	0.60 (0.36) *P* = 0.10

N, number; SUA, serum uric acid.

Plus-minus values are means ± SEM.

^*^*1, *2 and *3 represent haplotypes of two dysfunctional variants (Q126X and Q141K) as previously reported. Detailed information on *ABCG2* haplotypes is also shown in Table 1.

^†^106 controls were matched for sex- and body-mass index to 106 hemodialysis patients and selected from health examination participants in J-MICC Study.

^‡^β means regression coefficient.

^§^*P* values were obtained by multiple regression analysis including ABCG2 function and age in the model.

^||^*P* values were obtained by linear regression analysis.

**Table 3:** Dehydration in acute gastroenteritis patients for each ABCG2 function.

Estimated ABCG2 function (Diplotype of Q126X and Q141K)*	Number			*P* value‡
	Acute gastroenteritis	Dehydration†		
		−	+	
Full function (*1/*1)	29	23	6	
3/4 function (*1/*2)	30	20	10	
≤1/2 function (*1/*3, *2/*2, *2/*3 or *3/*3)	8	6	2	
Total	67	49	18	0.50

^*^*1, *2 and *3 represent haplotypes of two dysfunctional variants (Q126X and Q141K). Detailed information on *ABCG2* haplotypes is also shown in Table 1.

^†^“−” means minimal or no dehydration and “+” means mild to moderate or severe dehydration evaluated according to the criteria recommended by the Center for Disease Control (CDC).

^‡^*P* values were obtained by Cochran-Armitage test.
