# Supplementary material for: Hyperuricemia in acute gastroenteritis is caused by decreased urate excretion via ABCG2
Source: Sci Rep. 2016 Aug 30;6:31003. doi: 10.1038/srep31003 (PMC5004129; doi:10.1038/srep31003)
Supplement: Supplementary Information [file srep31003-s1.doc]

SUPPLEMENTARY INFORMATION

**Hyperuricemia in acute gastroenteritis is caused by decreased urate excretion *via* ABCG2**

Hirotaka Matsuo*, Tomoyuki Tsunoda*, Keiko Ooyama*, Masayuki Sakiyama*, Tsuyoshi Sogo, Tappei Takada, Akio Nakashima, Akiyoshi Nakayama,

Makoto Kawaguchi, Toshihide Higashino, Kenji Wakai, Hiroshi Ooyama,

Ryota Hokari, Hiroshi Suzuki, Kimiyoshi Ichida, Ayano Inui, Shin Fujimori & Nariyoshi Shinomiya

*These authors contributed equally.

Correspondence to H.M. (email: hmatsuo@ndmc.ac.jp).

Supplementary Table 1 | Haplotype frequency of two *ABCG2* variants

Supplementary Table 2 | Degree of dehydration and SUA in acute period of gastroenteritis patients

Supplementary Table 3 | Clinical characteristics of participants

**Supplementary Table 1. Haplotype frequency of two *ABCG2* variants.**

| **rs72552713 (Q126X)** | **rs2231142 (Q141K)** | **Haplotype frequency** |
| --- | --- | --- |
| C | C | 0.692 |
| C | A | 0.285 |
| T | C | 0.023 |
| T | A | 0^*^ |

Haplotype frequency has been calculated using the genotype data of all participants (106 hemodialysis patients, 106 health examination participants and 67 acute gastroenteritis patients) in this study.

^*^“T-A” haplotype could not exist, which means that there is no simultaneous presence of the minor allele of Q126X and Q141K in one haplotype.

**Supplementary Table 2. Degree of dehydration and SUA in acute period of gastroenteritis patients.**

| **Degree of dehydration^*^** | **Number (%)** | **SUA (mg/dl)** | ***P* value**^†^ |
| --- | --- | --- | --- |
| Minimal or no dehydration | 49 (73.1%) | 8.0 ± 0.4 |  |
| Mild to moderate dehydration | 18 (26.9%) | 11.0 ± 1.0 |  |
| Severe dehydration | 0 (0%) | - |  |
| Total | 67 (100%) | 8.8 ± 0.4 | 1.6 × 10^-3^ |

SUA, serum uric acid.

Plus-minus values are means ± SEM.

^*^Severity of dehydration was evaluated according to the criteria recommended by the Center for Disease Control (CDC).

^†^*P* values were obtained by linear regression analysis.

**Supplementary Table 3. Clinical characteristics of participants.**

|  | **Hemodialysis**  **patients** | **Health examination participants^*^** | | **Acute gastroenteritis patients** |
| --- | --- | --- | --- | --- |
| Number | 106 | 106 | 67 | |
| Sex, Male/Female | 73 (68.9%) / 33 (31.1%) | 73 (68.9%) / 33 (31.1%) | 34 (50.7%) / 33 (49.3%) | |
| Age (year) | 65.4 ± 13.0 | 59.0 ± 8.3 | 3.8 ± 3.1 | |
| Body mass index (kg/m^2^) | 21.9 ± 4.1 | 21.9 ± 4.1 | 15.3 ± 1.4 | |

Plus-minus values are means ± SD.

^*^106 health examination participants were matched for sex and body-mass index to 106 hemodialysis patients and selected from J-MICC Study.
